# Peppermint Essential Oil-Doped Hydroxyapatite Nanoparticles with Antimicrobial Properties

**DOI:** 10.3390/molecules24112169

**Published:** 2019-06-09

**Authors:** Monica Luminita Badea, Simona Liliana Iconaru, Andreea Groza, Mariana Carmen Chifiriuc, Mircea Beuran, Daniela Predoi

**Affiliations:** 1 Faculty of Horticulture, University of Agronomic Sciences and Veterinary Medicine, 59 Mărăşti Blvd., 011464 Bucharest, Romania; badea.artemisia@gmail.com; 2 Multifunctional Materials and Structures Laboratory, National Institute of Materials Physics, Atomistilor Street, No. 405A, P.O. Box MG 07, 077125 Magurele, Romania; simonaiconaru@gmail.com; 3 Low Temperature Plasma Laboratory, National Institute for Laser, Plasma and Radiation Physics, 409 Atomistilor Street, P.O. Box MG 36, 077125 Magurele, Romania; andreeagroza75@gmail.com; 4 Microbiology Department, Faculty of Biology, University of Bucharest, 1–3 Portocalelor Lane, 77206 Bucharest, Romania; carmen.chifiriuc@gmail.com; 5 Earth, Environmental and Life Sciences Section, Research Institute of the University of Bucharest (ICUB), 91-95 Splaiul Independentei, 050095 Bucharest, Romania; 6 Department of Surgery, Carol Davila University of Medicine and Pharmacy, 8 Eroii Sanitari, Sector 5, 050474 Bucharest, Romania; beuranmircea@gmail.com; 7 Emergency Hospital Floreasca Bucharest, 8 Calea Floresca, 014461 Bucharest, Romania

**Keywords:** hydroxyapatite, peppermint essential oil, antimicrobial properties

## Abstract

This study aimed at developing an antimicrobial material based on hydroxyapatite (HAp) and peppermint essential oil (P-EO) in order to stimulate the antimicrobial activity of hydroxyapatite. The molecular spectral features and morphology of the P-EO, HAp and hydroxyapatite coated with peppermint essential oil (HAp-P) were analyzed using Fourier-transform infrared (FTIR) spectroscopy and scanning electron microscopy (SEM). The coating of the HAp with the P-EO did not affect the ellipsoidal shape of the nanoparticles. The overlapping of IR bands of P-EO and HAp in the HAp-P spectrum determined the formation of the broad molecular bands that were observed in the spectral regions of 400–1000 cm^−1^ and 1000–1200 cm^−1^. The antibacterial activity of the P-EO, HAp and HAp-P were also tested against different Gram-positive bacteria (methicillin-resistant *Staphylococcus aureus* (MRSA) 388, *S. aureus* ATCC 25923, *S. aureus* ATCC 6538, *E. faecium* DSM 13590), Gram-negative bacteria (*Escherichia coli* ATCC 25922, *E. coli* C5, *P. aeruginosa* ATCC 27853, *P. aeruginosa* ATCC 9027) and a fungal strain of *Candida parapsilosis*. The results of the present study revealed that the antimicrobial activity of HAp-P increased significantly over that of HAp.

## 1. Introduction

Aromatic herbs like peppermint, lavender, basil, oregano, and thyme have wide applications in the pharmaceutical or food industries due to their positive health effects [1,2,3,4]. The essential oils extracted from these aromatic plants are of great interest, especially due to their antifungal, antibacterial, and antioxidant properties [1].

The essential oils extracted from aromatic plants have the following bioactive components in their chemical composition: monoterpenes, monoterpenoids, and phenylpropanoids. Their concentrations in the extracted oils depend strongly on the soil, climatic, and geographical conditions [1,2,3,4] of the herbal culture growing.

P-EO has proven its utility against the following pathologies: ulcers, obesity, cancer, diabetes, gastrointestinal complications, and immunomodulation [5]. In particular, P-EOs possess antiviral activities that help the immune system fight viruses, including Herpes simplex virus 1 (HSV-1) and Herpes simplex virus 2 (HSV-2) [6,7,8]. In some previous studies, P-EO has been reported to exhibit a strong antibacterial effect against *Enterococcus faecium* ATCC10541, *Salmonella choleraesuis, Staphylococcus aureus,* and *Bacillus subtilis.* [9,10,11,12,13,14]. The antifungal properties of P-EO were pointed out against *Candida albicans, Aspergillusalbus* and *dermatophytic* fungi [15].

The latest approaches in the treatment of pathogenic viruses frequently imply the combination of phototherapies that use the benefits of essential oils with conventional antiviral therapies [5,6,7,8,9,10,11,12,13,14,15].

Nowadays, the healthcare and biomedical industries frequently use biomaterials with proper physical, chemical and antimicrobial properties for improving the antibacterial or antifungal properties of their products. Ceramic biomaterials play an important role, as they are used as coatings for biomedical implants. Hydroxyapatite ((Ca_10_(PO_4_)_6_(OH)_2_) is the best well-known biochemical compound with biocompatible properties towards the human tissue that belongs to the calcium phosphate family [16,17].

*Mentha* species are the most widely used medicinal herbs due to their chemical constituents, menthol and menthone. Usually, peppermint (*Mentha piperita*) essential oils are used as remedies in coughs, colds, mouth sinuses, pain relief, and headaches, as well as being used for skin problems, digestive affections, and as a muscle relaxant, and so on [18,19]. The pharmacological activities of P-EOs, such as its antiviral activity against influenza, herpes, and other viruses [20], as well as its antihelmentic effect [21], was reported by Kerman and Kucera in their paper regarding “Antiviral Substances in Plants of the Mint Family. Peppermint and other Mint Plants” [20] and by Girme et al. in their studies involving “Comparative In Vitro Anthelmintic Activity of Mentha piperita and Lantana camara from Western India” [21]. Based on the literature survey, menthol is generally reported as the main component of P-EOs [22,23,24,25], while menthone and limonene are always present in meaningful concentrations [22,25]. According to previous studies, P-EOs are mainly composed of menthol in the range of 29%–48%, with menthone in concentrations varying from 20% to 31%, menthofuran with concentrations around 6.8%, and menthyl acetate in concentrations from 3% to 10% [26]. Even though there are several papers reporting the chemical composition of P-EOs, the chemistry of essential oils is very complex and varies significantly. The relative concentrations of chemical constituents vary depending on the climate, geographic location, time of the harvesting, drying conditions, extraction method, and so on [27,28,29,30,31].

In our previous studies [32,33], we have analyzed the changes induced by the adsorption of lavender and basil essential oils on the physico-chemical and morphological properties of hydroxyapatite nanoparticles. These studies indicated a better adsorption of the lavender essential oil on the surface of hydroxyapatite nanoparticles, and antibacterial tests proved their better antibacterial activity against *S. aureus*, *E coli*, and MRSA bacterial strains in comparison with the hydroxyapatite nanoparticles covered with basil essential oil.

Our research presented in this study focused on materials in the form of solutions that could be used in various medical applications. In the process of covering some implants, solutions might be more effective. On the other hand, solutions could offer a wider range of applicability in the pharmaceutical, medical, chemical, and even food industries. Moreover, these solutions may, in combination with other solutions, help to reduce the spread of diseases and, implicitly, improve the health of populations in all types of scenarios.

The aim of the present study was to analyze of the influence of P-EO adsorbed on the surface of hydroxyapatite nanoparticles and their morphological, physicochemical, and antimicrobial properties. Scanning electron microscopy (SEM) was used to determine the morphology of HAp-P in comparison with HAp. The molecular spectral analysis of the HAp-P samples was performed using Fourier-transform infrared spectroscopy. The antimicrobial activity against different Gram-positive (methicillin-resistant *S. aureus* (MRSA) 388, *S. aureus* ATCC 25923, *S. aureus* ATCC 6538, *E. faecium* DSM 13590) bacteria, Gram-negative bacteria (*E. coli* ATCC 25922, *E. coli* C5, *P. aeruginosa* ATCC 27853, *P. aeruginosa* ATCC 9027), and a fungal strain (*Candida parapsilosis*) was also investigated.

## 2. Results and Discussions

The P-EOs obtained from dried *M. piperita* plants were qualitatively and quantitatively analyzed by GC–MS. The results represented 97.37% of the essential oil, and the primarily chemical compounds that were identified were menthol (35.21%), menthone (21.56%), menthyl acetate (6.90%), piperitone (5.60%), limonene (5.40%), and 1,8-cineole (5.30%). The results also highlighted the presence of germacrene-D (3.10%), myrcene (2.80%), neo-menthol (2%), menthofuran (1.50%), and β-pinene (1.050%). Other compounds specific to P-EOs such as 3-octanol, linalool, α-thujene, α-pinene, camphene, sabinene, α-terpinene, α-terpinolene, mentholacetate, pulegone, iso-pulegol, neosiomenthol, terpinene-4-ol, α-terpinoel, cis-β-ocimene, cis-sabinene-hydrate, and p-cymene were also identified in concentrations lower than 1%. These results are in good agreement with the results obtained by Iscan et al. [22], Schmidt et al. [31], and Mahboubi and Kazempour [18], who also reported menthol and menthone being the principal constituents in the P-EOs investigated in their studies. The data obtained by GC studies emphasized that the most abundant chemical constituents of the P-EOs investigated in our study were monoterpenes. The chemical composition of essential oils is always important, and has a high involvement in the antimicrobial properties of EOs.

**Figure 1:**
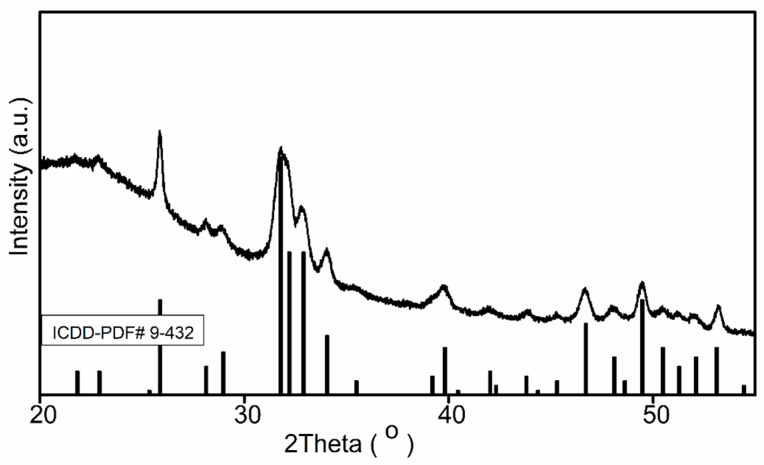
XRD pattern of HAp powder.

Morphological and structural studies were carried out by SEM, XRD, and FTIR, the HAp and HAp-P powders were obtained by centrifuging the solutions. The resulting precipitation after centrifugation was dried at 30 °C in an oven. Phase identification of the HAp powders was conducted by XRD analysis. Figure 1 shows the crystalline structures of HAp powders obtained by centrifuging the solutions. All diffraction peaks were assigned to the pure Hap, in agreement with ICDD-PDF #9-432 and literature [34,35], and no further phases were identified. The main diffraction peaks at 2θ (°) 25.88, 31.78, 32.90, 34.06, 39.82, 46.71 and 49.47 were assigned to the HAp [36,37].

These new solutions based on HAp and P-EO could be a problem-solving development in the medical field by integrating innovative technologies into the medical environment. This could be an important part of keeping medical infrastructure cleaner.

**Figure 2:**
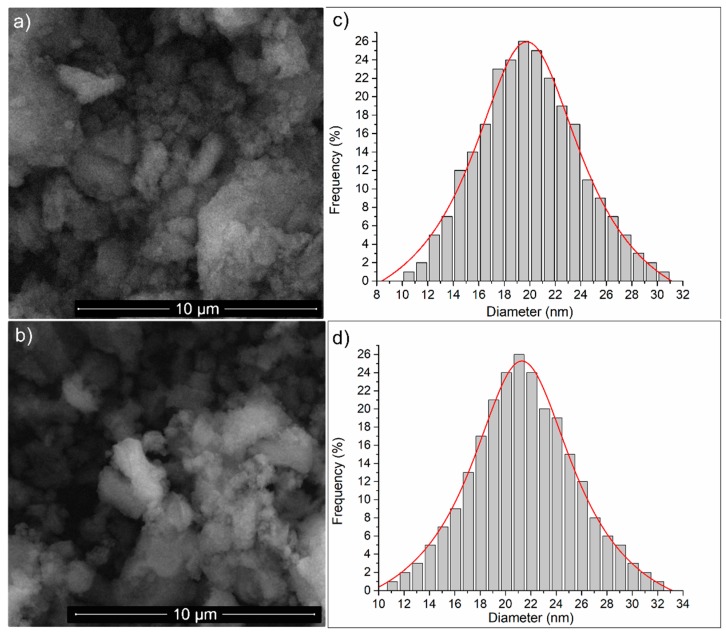
SEM images of (**a**) HAp and (**b**) HAp-P, and particle size distributions of (**c**) HAp and (**d**) HAp-P samples.

The morphology of the samples were investigated by SEM. The granular structure of the HAp and HAp-P samples is shown in Figure 2. It can be observed that the HAp nanoparticle agglomerates having ellipsoidal shapes. The SEM image of the HAp-P sample (Figure 2b) indicates that the morphology of the HAp nanoparticles (Figure 2a) did not exhibit any significant changes, and their nanometric size and ellipsoidal shape were maintained after the adsorption of the P-EO on the HAp nanoparticles. Furthermore, a size distribution was obtained for the HAp (Figure 2c) and HAp-P (Figure 2d) samples. The size distribution was done using particles from four areas of 2.5 × 2.5 µm in each SEM image. The results of the particle size distributions revealed that the size of the HAp nanoparticles were approximately 19.56 ± 3 nm and the size of the HAp-P approximately 21.10 ± 5 nm. The SEM studies emphasized that the adsorption of P-EO on the surface of HAp had a small effect on the size of the particles, and had no effect on their ellipsoidal shape.

**Figure 3:**
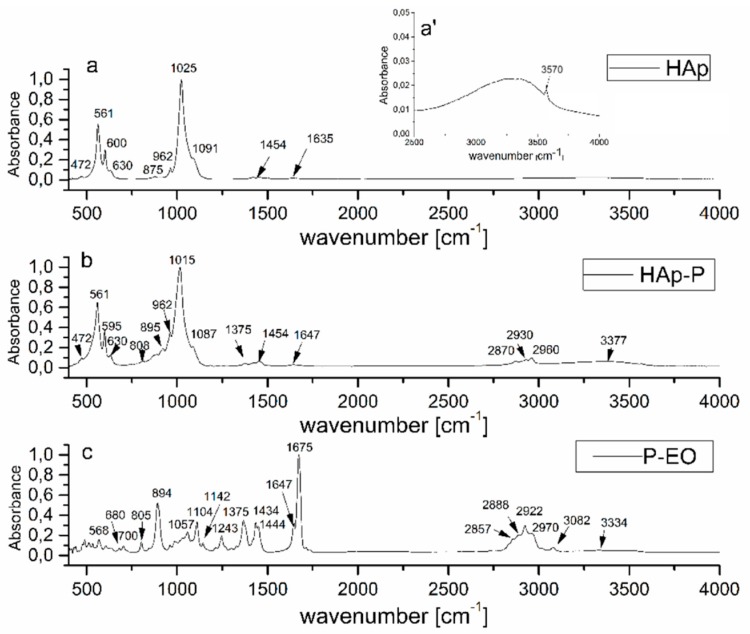
FTIR spectrum of (**a**) HAp, (**b**) HAp-P (**c**) and P-EO samples; FTIR spectra from of HAp sample from 2500 to 4000 cm^−1^ (**a’**).

IR absorbance spectra of HAp, HAp-P, and P-EO are presented in Figure 3 in the 400–4000 cm^−1^ range. The spectra exhibited bands of HAp (Figure 3a,b) and P-EO (Figure 3b,c) ascribed to [HPO_4_]^2−^ (at 875 cm^−1^) and [CO3]^2−^ (at 1454 cm^−1^) impurity ions that were the result of a chemical synthesis process of HAp nanoparticles nearly visible in Figure 3a–b [38,39,40]. The peak broad at 1636 cm^−1^ is assigned to the ν_2_ bending mode of water. The typical HAp structure was highlighted by the symmetric P-O stretching mode that was observed at 962 cm^−1^ (ν_1_). The hydroxyl group was evidenced by the bands from 630 cm^−1^ (ν_L_) and 3570 cm^−1^ (ν_S_). The band at 3570 cm^−1^ is clearly observed in Figure 3a’ [17,40,41,42,43].

The [PO_4_]^3−^ vibrational groups of the apatitic structure present the fundamental vibrational modes at 472 cm^−1^ (ν_2_), 561 (ν_4_), 600 (ν_4_), 1025, and 1091 cm^−1^ (ν_3_) [42,43].

The IR absorption bands of the P-EO sample are shown in the IR spectrum from Figure 3c. The peak at 3082 is attributed to aromatic C-H bonds. According to N. Kasiri et al. [44], the peaks at 3000–2850 cm^−1^ are the result of C-H stretching. The band at 2970 cm^−1^ is due to asymmetric CH_3_ stretching, while the band at 2926 cm^−1^ is allocated to asymmetric CH_2_ stretching [45]. The wavenumbers from 1057, 1108, and 1243 cm^−1^ belong to C-O stretching vibrations, while the absorption bands identified at 700, 805, 894, 1364, 1434, and 1444 cm^−1^ are caused by vibrations of the C-H bonds in the peppermint structure [46]. In the spectrum of the HAp-P from Figure 3b, IR bands characteristic of P-EO can be identified (marked in Figure 3c). In the spectral regions of 400–1000 and 1000–1200 cm^−1^, the overlapping of P-EO and HAp IR bands determined the formation of the broad molecular bands observed in Figure 3b [47,48,49,50,51]. Finally, it should be noted that the absorption bands at 917, 1283, 2430, 3320, 3487, 3561, and 3581 that distinguish the formation of octacalcium phosphate are not observed in the HAp and HAp-P spectra, and certify the HAp stoichiometry (Ca/P ratio = 1.67). In conformity with previous studies presented by J. Allene et al. [52] and T. Heinze [53], compounds with high flexion due to the (Ca^2+)^ group can interact with the (-OH) of the polymers chains. Therefore, compounds such as hydroxyapatite can interact with the (-OH) from the essential oil structure.

The results of the qualitative antimicrobial properties of P-EO and HAp-P are presented in Table 1. The results of the antimicrobial qualitative assays revealed that the peppermint had a significant inhibition effect on the microbial growth of the tested microorganisms, with zone inhibition diameters ranging from 6 to 22 mm. The solvent, DMSO, did not affect the growth on solid media of any of the tested microbial strains. Moreover, HAp had no inhibitory effect on the growth of the tested microorganisms. The most pronounced inhibitory effect was observed in the case of *E. coli* tested strains (*E. coli* ATCC 25922 and *E. coli* C5). The diameter of the inhibition growth zone were 22 and 20 mm in the case of P-EO samples. The HAp-P samples produced a smaller inhibition zone on the *E. coli* strains, 8 mm for *E. coli* ATCC 25922 and 7 mm for *E. coli* C5. A notable inhibitory effect on *S. aureus* bacterial strains has also been observed. The inhibition zone of the HAp-P sample for *S. aureus* ATCC 25923 was 10 mm, while for *S. aureus* ATCC 6538 was 7 mm.

The investigation of the antimicrobial properties of P-EO and HAp-P was carried out using 96 well plates with two-fold dilutions. The values for the minimum inhibitory concentration (MIC) an minimum bactericidal concentration (MBC) obtained for DMSO, HAp, P-EO, and HAp-P are presented in Table 2. The MIC values of the P-EO obtained against the tested microorganisms ranged from 15.62 to 62.5 µL/mL. The most sensitive microorganism to the tested samples was *C. parapsilosis* ATCC 22019 (MIC value 15.62 µL/mL). The results obtained from the measurements of MIC and MBC indicated that HAp-P exhibited a good antibacterial activity against tested bacteria, and the tenderness was as follows: *P. aeruginosa* > *C. parapsilosis* > *E. faecium* > *E. coli* > *S. aureus* > MRSA. The data suggest that the DMSO solvent did not affect bacterial growth.

**Figure 4:**
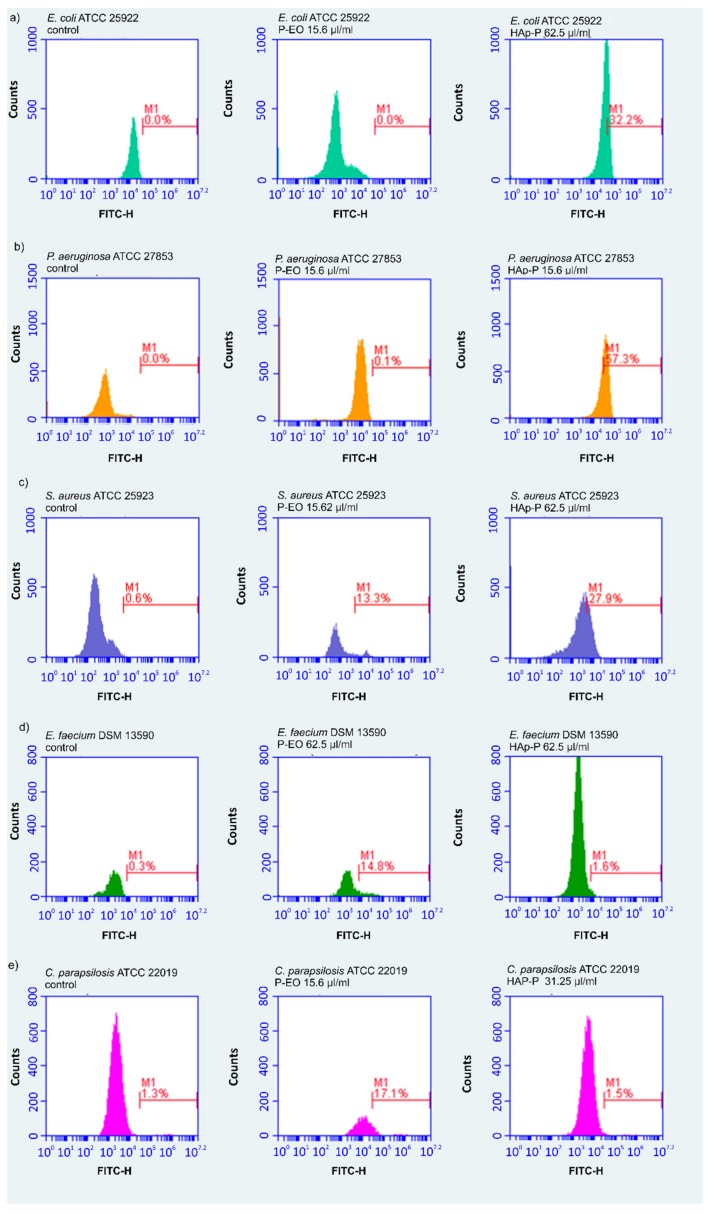
DiBAC4(3) staining (log fluorescence) of (**a**) *Escherichia coli* ATCC 25922, (**b**) *Pseudomonas aeruginosa* ATCC 27853, (**c**) *Staphylococcus aureus* ATCC 25923, (**d**) *Enterococcus faecium* DSM 13590 and (**e**) *Candida parapsilosis* ATCC 22019. Part of the fluorescent depolarized bacteria with increased green fluorescence caused by an accumulation of the dye inside the microbial cell, expressed in percentages.

The influence of the P-EO and HAp-P on the microbial membrane of the tested microorganism was investigated by flow cytometry (Figure 4). DiBAC4(3) is a green fluorescent dye and also a potential membrane reporter due to the fact that accumulates inside depolarized microbial cells cause an increase of green fluorescence. The flow cytometry studies determined the percentage of depolarized microbial cells, and the results of the flow cytometric tests presented in Table 3 emphasized that there was an increase in the green fluorescence of the microbial cells (*S. aureus* ATCC 25923, *E. faecium* DSM 13590, *C. parapsilosis* ATCC 22019) treated with the P-EO and HAp-P compared with the control ones (untreated microbial cells). On the other hand, an increase in the green fluorescence of *E. coli* ATCC 25922 and *P. aeruginosa* ATCC 27853 microbial cells treated with the P-EO and HAp-P versus the control was observed only for HAp-P.

Essential oil extracted from peppermint contains effective active constituents that are responsible for eliminating bacterial pathogens. P-EO and HAp-P showed significant antimicrobial activity because compounds such as the menthol and menthone present in these samples acted on the cell membrane, causing substantial morphological damage and leading to destabilization of the microbial membrane [54,55]. According to D. Lopes-Lutz et al. [56], the presence of other antimicrobial constituents such as limonene, neomenthol, carveone, or 1,8-cineole mixed with other minor constitutes like α-pinene, menthofuran, β-fenchyl alcohol, trans caryophyllene, β- cubebene, and α- terpinene could be involved in improving the whole antimicrobial activity of materials based on essential oils. Deans and Baratta [57], in previous studies on the antimicrobial and antioxidant properties of some essential oils, reported similar results regarding the biological activity of P-EO against pathogenic bacteria. Valsaraj et al. [58] showed that essential oil extracted from peppermint leaves shows antibacterial activity only in ethyl acetate, petroleum ether, colloform, menthol, and aqueous extracts. On the other hand, antibacterial activity is dependent both on the dose and on the part of the plant (root, stem, leaves) from which the essential oil [30] has been extracted.

The combination of hydroxyapatite with P-EO could be used in different coatings that could be used in different implants to reduce the effective doses of antibiotics, as well as the side effects and risks associated with them. On the other hand, HAp-P-coated implants could help reduce postoperative infections that can lead to major risks to patients’ lives.

## 3. Materials and Methods

### 3.1. Sample Preparation

The essential oil (EO) used in this study was P-EO. The peppermint plants (*M. piperita*) were harvested just before their full bloom, during summer (second week of July 2017), from an independent farm situated in southeast Romania. After being harvested, the peppermint plants were dried at room temperature, packed in paper bags, and stored in a cool and dry place. The P-EO was obtained by hydrodistillation from dried plants.

Hydroxyapatite Ca_10_(PO_4_)_6_(OH)_2_ solutions (HAp) were obtained by an adapted coprecipitation method [17] using as precursors calcium nitrate (Ca(NO_3_)_2_∙4H_2_O, Sigma Aldrich, St. Louis, MO, USA), ammonium hydrogen phosphate ((NH_4_)_2_HPO_4_; Wako Pure Chemical Industries Ltd., Richmond, VA, USA), ammonium hydroxide (NH_4_OH, Wako Pure Chemical Industries Ltd., Richmond, VA, USA), and double-distilled water. The synthesis was done maintaining the molar ratio Ca:P = 1:67 [17]. The P-EO was added to the final HAp solution and the mixture was stirred for 24 h at room temperature. The final product, HAp-P, was analyzed by different techniques. An antimicrobial activity evaluation of the solutions was also conducted.

### 3.2. Characterizations

#### Structural and Morphological Characterizations

The chemical composition of the P-EO samples was analyzed by gas chromatography (GC) using a Perkin Elmer gas chromatographer (PerkinElmer Inc, Waltham, MA, USA) equipped with a flame ionization detector (FID) (PerkinElmer Inc, Waltham, MA, USA). The working conditions were as previously described in Predoi et al. [33]. Furthermore, a GC–MS analysis of the P-EOs was done with the aid of a PekinElmer Turbomass Quadrupole mass spectrometer (PerkinElmer Inc, Waltham, MA, USA), as previously described in Predoi et al. [33]. The compounds were identified with the NIST and Wiley Registry 8 Edition mass database, n-alkalene (C_9_-C_22_) hydrocarbon series (Nile, Italy), and the mass spectra found in the literature [59,60] as comparative elements. The results of the chemical composition were presented as relative area percent (%).

The XRD measurements were used for investigating the structure of the HAp and HAp-P samples. A Bruker D8 Advance diffractometer (Bruker, Karlsruhe, Germany) with a nickel-filtered Cu Kα (λ = 1.5418 Å) radiation in the 2θ ranging from 20° to 80° was used in these studies.

The surface morphology of the HAp, and HAp-P samples was analyzed by scanning electron microscopy (SEM) using a HITACHI S4500 microscope. The HAp and HAp-P nanoparticles were carefully placed on the holder using a conductive carbon tape with double adhesion. The SEM images of the samples were acquired with a 5 kV electron acceleration voltage and an Everhart-Thornley detector (ETD).

Analysis of the molecular spectral features of P-EO, Hap, and HAp-P samples was performed by FTIR measurements using a PerkinElmer spectrometer (Waltham, MS, USA) SP-100 with a 4 cm^−1^ resolution. The transmission IR spectra were acquired using an attenuated total reflection (ATR) accessory and were converted into absorption spectra using SPECTRUM software (Version 6.4.1 PerkinElmer, Waltham, MS, USA) following the A = log (1/T) formula. The spectra were then normalized from 0 to 1 [32,61].

### 3.3. Antimicrobial Assays

#### 3.3.1. Microbial Strains and Culture

The antimicrobial properties of the P-EO and HAp-P were assessed using both reference and clinical microbial strains from the Department of Microbiology, Faculty of Biology, University of Bucharest. The tested microbial strains were Gram-negative bacteria (*E. coli* ATCC 25922, *E. coli C5* (carbapenemase producer strain), *P. aeruginosa* ATCC 27853, *P. aeruginosa* ATCC 9027) Gram-positive bacteria (*S. aureus* ATCC 25923, *S. aureus* ATCC 6538, *E. faecium* DSM 13590), and fungi (*C. parapsilosis* ATCC 22019).

#### 3.3.2. Agar Diffusion Method

The qualitative antimicrobial assay of the P-EO and HAp-P was investigated using an adapted agar diffusion method [62,63,64,65]. Microbial suspension corresponding to 0.5 McFarland, obtained from 18–24 h cultures of the tested microorganism, were inoculated on Mueller-Hinton (MH) agar plates. Furthermore, the P-EO and HAp-P were deposited on the agar surface and incubated at 37 °C in aerobic conditions for 24 h. After 24 h of incubation, the diameters of the inhibition growth zones were measured.

#### 3.3.3. Broth Micro-Dilution Assay

The antimicrobial properties of P-EO and HAp-P were investigated using the broth micro-dilution assay. Serial two-fold dilutions of the samples solubilized in dimethyl sulfoxide (DMSO) were done in 96 well plates in MHB (Muller Hinton Broth). Furthermore, microbial suspensions with a density of 10^6^ CFU mL^−1^ were also prepared from 24 h solid cultures. The microbial suspensions were then inoculated on each microtiter well containing the two-fold dilutions of P-EO and HAp-P. A sterility control was added with 100 μL of MHB and the antimicrobial properties of the DMSO were also tested. The experiments were done in triplicate. The wells were incubated for 18–24 h in aerobic conditions, at 37 °C. After incubation, the minimum inhibitory concentration (MIC) values and the minimum bactericidal concentration (MBC) values were determined using the conventional plating method.

#### 3.3.4. Flow Cytometry Testing

Studies regarding the effect of the P-EO and HAp-P samples against the microbial membrane were conducted by flow cytometry using a sensitive fluorescent dye, DiBAC4(3). The microbial strains were cultivated in the presence of the tested samples for 24 h at 37 °C. After 24 h, 0.5 μg/mL of DiBAC4(3) (Invitrogen/Life technologies, Carlsbad, CA, USA) was used to stain the microbial strains. The positive control was represented by an untreated microbial cell suspension. After 30 min of incubation with the DiBAC4(3), the intensity of fluorescence (IF) was measured using an Accuri C6 plus flow cytometer (Becton Dickinson, Biosciences, (San Jose, CA, USA)) in channel of fluorescence fluorescein isothiocyanate (FITC) (filters 530/30 nm).

### 3.4. Statistical Analyses

The biological experiments were done in triplicate and the results were represented as mean ± standard deviation (SD). The statistical analysis was performed using a Student’s *t*-test, and only values of *p* < 0.05 were taken into consideration.

## 4. Conclusions

This is the first study developed on hydroxyapatite coated with peppermint essential oil. The GS-MS studies revealed that the main chemical constituents of the P-EOs were menthol and menthone. The molecular spectral features and morphology of the P-EO, HAp and HAp-P, were investigated. Moreover, this study presents for the first time the physicochemical and antimicrobial properties of HAp-P. FTIR studies have highlighted a high degree of absorption of P-EO in the HAp structure that were revealed by measured values of the P-EO IR absorption bands in the HAp-P spectrum. Furthermore, morphological studies have shown that HAp-P nanoparticles conserve the form of HAp nanoparticles. The results obtained from the MIC and MBC measurements showed that P-EO possessed a good antimicrobial activity against the tested microorganisms. On the other hand, MIC and MBC measurements indicated that the antimicrobial activity of HAp-P increased significantly from that of HAp following the adsorption of P-EO at the surface of HAp nanoparticles. The results of the present study are valuable for further investigations aimed at finding alternative solutions that could lead to a decrease in postoperative infections following implants.

## Figures and Tables

**Table 1:** The diameters of inhibition growth zones (mm).

Microbial Strains	P-EO	HAp-P	HAp	DMSO
*Escherichia coli* ATCC 25922	22 ± 0.2	8 ± 0.2	0 ± 0.1	0 ± 0.1
*Escherichia coli* C5	20 ± 0.3	7 ± 0.3	0 ± 0.1	0 ± 0.1
*Pseudomonas aeruginosa* ATCC 27853	10 ± 0.5	7 ± 0.5	0 ± 0.1	0 ± 0.1
*Pseudomonas aeruginosa* ATCC 9027	11 ± 0.3	6 ± 0.2	0 ± 0.1	0 ± 0.1
*Staphylococcus aureus* ATCC 25923	12 ± 0.3	10 ± 0.5	0 ± 0.1	0 ± 0.1
*Staphylococcus aureus* ATCC 6538	8 ± 0.2	7 ± 0.6	0 ± 0.1	0 ± 0.1
Methicillin-resistant Staphylococcus aureus (MRSA) 388	10 ± 0.5	0 ± 0.1	0 ± 0.1	0 ± 0.1
*Enterococcus faecium* DSM 13590	0 ± 0.1	0 ± 0.1	0 ± 0.1	0 ± 0.1
*Candida parapsilosis* ATCC 22019	0 ± 0.1	0 ± 0.1	0 ± 0.1	0 ± 0.1

**Table 2:** Minimum inhibitory concentration (MIC) and minimum bactericidal concentration (MBC) values obtained for DMSO, HAp, P-EO and HAp-P.

Microbial Strains	P-EO	HAp-P	HAp	DMSO
*Escherichia coli* ATCC 25922	31.25 ± 0.1 15.62 ± 0.3	250 ± 1.4 62.5 ± 0.4	>250 ± 1.5	>250 ± 1.6
*Escherichia coli* C5	31.25 ± 0.5 15.62 ± 0.5	250 ± 1.7 125 ± 1.9	>250 ± 1.5	>250 ± 1.8
*Pseudomonas aeruginosa* ATCC 27853	31.25 ± 0.5 15.62 ± 0.5	31.25 ± 0.5 15.62 ± 0.7	>250 ± 1.6	>250 ± 1.6
*Pseudomonas aeruginosa* ATCC 9027	31.25 ± 0.7 15.62 ± 0.5	31.25 ± 0.5 15.62 ± 0.3	>250 ± 1.9	>250 ± 1.5
*Staphylococcus aureus* ATCC 25923	31.25 ± 0.3 15.62 ± 0.4	250 ± 1.8 62.5 ± 0.5	>250 ± 1.2	>250 ± 1.9
*Staphylococcus aureus* ATCC 6538	31.25 ± 0.7 15.62 ± 0.3	250 ± 1.7 62.5 ± 0.9	>250 ± 1.5	>250 ± 1.3
Methicillin-resistant Staphylococcus aureus (MRSA) 388	31.25 ± 0.5 15.62 ± 0.5	250 ± 1.5 62.5 ± 0.7	>250 ± 1.8	>250 ± 1.6
*Enterococcus faecium* DSM 13590	62.5 ± 1.2 62.5 ± 0.9	125 ± 0.5 62.6 ± 0.2	>250 ± 1.4	>250 ± 1.7
*Candida parapsilosis* ATCC 22019	15.62 ± 0.3 15.62 ± 0.4	31.25 ± 0.6 31.25 ± 0.4	250 ± 1.5 125 ± 1.7	250 ± 1.5

**Table 3:** The percentage of depolarized bacterial cells treated with the selected plant EO and Hap and coated with plant EOs at MIC values.

Microbial Strains	P-EO	HAp-P	Control
*Escherichia coli* ATCC 25922	0.0%	32.2%	0.0%
*Pseudomonas aeruginosa aeruginosa* ATCC 27853	0.1%	57.3%	0.0%
*Staphylococcus aureus* ATCC 25923	13.3%	27.9%	0.6%
*Enterococcus faecium faecium* DSM 13590	14.8%	1.6%	0.3%
*Candida parapsilosis* ATCC 22019	17.1%	1.5%	1.3%
